# Association of physical activity with cancer incidence, mortality, and survival: a population-based study of men

**DOI:** 10.1038/sj.bjc.6604354

**Published:** 2008-05-27

**Authors:** N Orsini, C S Mantzoros, A Wolk

**Affiliations:** 1 Division of Nutritional Epidemiology, Institute of Environmental Medicine, Karolinska Institutet, Stockholm, Sweden; 2 Division of Endocrinology, Diabetes and Metabolism, Department of Medicine, Beth Israel Deaconess Medical Center, Harvard Medical School, Boston, MA, USA

**Keywords:** physical activity, cancer, mortality, men, cohort study

## Abstract

Within a population-based cohort study, 40 708 men aged 45–79 years followed from 1998 to 2004. After adjusting for potential confounders, we observed a strong inverse linear association between total daily physical activity (PA) and death from cancer (*n*=1153). For each increment of 4 metabolic equivalent (MET)-h day^−1^ of total PA (approximately 1 h daily of moderate effort) cancer incidence (*n*=3714) tended to be decreased by 2% and cancer mortality decreased significantly by 12% (95% confidence interval=6–18%). The 5-year survival after cancer among those men in the top quartile of total PA (77%) was significantly higher compared to the lowest quartile (69%). Compared to those men who hardly ever walked or biked, walking or bicycling an average of 30 min day^−1^ was associated with a 34% (18–47%) lower rate of cancer death and with improved cancer survival by 33% (14–47%). Incidence of cancer was 16% (2–28%) lower among those who walked or biked at least 60 min day^−1^. Our results suggest that higher levels of PA and the main component of active living, walking or bicycling are associated with reduced cancer incidence and mortality, as well as higher cancer survival.

Cohort studies have suggested that more physically active men, especially those involved in recreational and leisure-time activity, have lower cancer risk and mortality (Leon and Connett, 1991; Wannamethee *et al*, 1993; Kampert *et al*, 1996; Alfano *et al*, 2004; Hu *et al*, 2005; Leitzmann *et al*, 2007) though the reductions have tended to be moderate and not always significant (Leon and Connett, 1991).

In a cohort of current and former smokers drawn from a lung cancer chemoprevention trial, total hours per week spent in leisure-time activity was inversely associated with all cancer incidence and mortality, but the latter was significant only among younger men (⩽62 years) (Alfano *et al*, 2004). Another cohort study found that men with the highest physical activity (PA) level had 21% lower cancer mortality than those with the lowest level (Hu *et al*, 2005). In a large cohort, those following the PA recommendation (minimum of 30 min day^−1^ of moderate activity on most days) showed a significant 17% reduction in cancer mortality compared to inactive participants (Leitzmann *et al*, 2007).

Studies have not examined cancer incidence, mortality, and survival in relation to a quantitative index of total daily PA, incorporating the duration and intensity of various activities. Moreover, it remains unclear whether this follows a linear dose-response pattern, and how much daily activity reduces cancer (McTiernan *et al*, 1998; WCRF/AICR, 2007). In a cohort of middle-aged and elderly Swedish men, we therefore studied quantitatively total daily PA in relation to cancer risks.

## METHODS

In 1997–1998, all men (*n*=100 303) aged 45–79 years residing in Västmanland and Örebro counties (central Sweden) received an invitation to participate in the study. An accompanying questionnaire covered PA, current weight, height, education, cigarette smoking, alcoholic beverages, diabetes, family history of cancer, and other lifestyle factors. A total of 48 645 men returned the questionnaire. For the analysis, we excluded those who returned a blank questionnaire (*n*=92), who died before 1 January 1998 (*n*=55), and those who had cancer previously (information from linkage to the National inpatient register at the National Board of Health and Welfare) (*n*=2592). We also excluded heavy manual workers (*n*=5198) because overall mortality from cancer has been found to be significantly higher among men with manual occupations (Rosengren and Wilhelmsen, 2004). After these exclusions, the study cohort comprised 40, 708 men which well represents the whole Swedish male population aged 45–79 years in terms of age, educational level, and prevalence of overweight (Norman *et al*, 2002).

Information on PA was collected using five questions (occupation, housework, walking/bicycling, active leisure-time exercise, inactive leisure-time watching TV/reading) about duration and intensity of usual PA in the past year. In the questionnaire there were six predefined PA levels for occupational activity (from mostly sitting down to heavy manual labour) and five to six predefined categories for time spent on different activities: walking/bicycling (from hardly ever to more than 1.5 h day^−1^), home/household work (less than 1 h to more than 8 h day^−1^), inactive leisure-time watching TV/reading (from 2 h day^−1^ or less to 5 h day^−1^ or more), and active leisure time exercising (from less than 1 h to more than 5 h week^−1^). There was also an open question about the number of sleeping hours per day. To calculate the activity score of specific type of activity, its intensity defined as metabolic equivalents (MET, kcal kg^−1^ h^−1^) was multiplied by reported duration (hours) (Ainsworth *et al*, 2000). We then estimated the total daily activity (24 h) score by adding all specific types of activities together. The PA questions were validated using two 7-days of activity records performed 6 months apart in a group of Swedish men 44–78 years of age and were shown to correlate well with total PA; Spearman correlation coefficient between questionnaire and PA records was 0.6. The reproducibility of total PA as reflected by Spearman correlation coefficient between the first and second (6 months later) self-administered questionnaires was 0.65 (Norman *et al*, 2001).

Date of death was ascertained through the Swedish Death Register at Statistic Sweden and cancers by computerised linkage with the National Swedish Cancer Register and the Regional Cancer Register covering the study area, both virtually complete (Mattsson and Wallgren, 1984). Both were classified by the International Classification of Diseases (ICD-10, C00-C97). Follow-up lasted 7 years from 1 January 1998 to 2004.

### Statistical analysis

Baseline age-standardised characteristics were presented by quartiles of total PA in MET-h day^−1^. The Cox-proportional hazards model was used to estimate rate ratios (RRs) and 95% confidence intervals (CIs) for total PA (continuous). We treated total PA both as quartiles and as continuous variables, which allows a more flexible and efficient use of available information. In our main analysis the end points were cancer incidence and cancer mortality. Each participant accrued follow-up time from 1 January 1998 until the date of cancer diagnosis (for incidence) or cancer death (for mortality), death from any cause, or study end on 31 December 2004, whichever came first. Cancer survival was also studied from diagnosis until death or study end, whichever occurred first.

We adjusted for age (continuous), body mass index (BMI, weight in kilograms divided by height in metres squared, kg m^−2^, as continuous) and other potential confounders, including smoking status and pack-years of smoking (never, former <20 pack-years, 20–39 pack-years, ⩾40 pack-years; current <20 pack-years, 20–39 pack-years, ⩾40 pack-years), alcohol (current, former, never drinker), educational level (less than high school, high school graduate, and more than high school), diabetes (yes, no), and parental history of cancer (yes, no, not known).

We checked whether the proportional hazard assumption was reasonable in the multivariate models by calculating scaled Schoenfeld's residuals, regressed against survival time, and tested for a nonzero slope; there was no evidence of departure. We used restricted cubic spline Cox regression to flexibly model the association between total PA (continuous) and cancer incidence and mortality rates. Three knot positions were specified for total PA in MET-h day^−1^ corresponding to the 25th, 50th, and 75th percentile of the observations.

A total of 75% of participants had complete data on specific types of activity (five questions) included in the total PA score, 13% had one missing value (one of five questions not answered), 3% had two missing values, 3% had three to four missing values, and 6% had five missing values; most of the missing answers regarded occupational activity because many participants was retired. The proportion of missing covariate data was 5% for BMI, and less than 5% for smoking, alcohol, educational level, history of diabetes, and parental history of cancer.

A complete analysis was based on 28 880 men in the analytic cohort, and 727 cancer deaths. To evaluate a potential effect of missing values, we used multivariate imputation by chained equations (MICE) to obtain five imputed data sets of the analytic cohort (van Buuren *et al*, 1999; Royston, 2004). The RRs so obtained were pooled together by using the Rubin's rule to obtain valid statistical inferences (Rubin and Schenker, 1986). We examined PA in relation to cancer mortality by age (⩽65, >65 years), BMI (<25, ⩾25 kg m^−2^), and smoking status (never/former, and current) and tested the statistical significance of the interactions with the Wald test.

In our secondary analysis among 2551 men with cancer and complete information about PA and confounders, 598 men died from cancer. Cumulative survivor functions for low (bottom), medium (second and third), and high (top quartile) total PA level were estimated using a multivariable Cox regression model and plotted *vs* time since cancer diagnosis. All reported *P*-values are two-sided; *P*-values of less than 0.05 were considered statistically significant. All statistical analyses were performed with Stata, version 9.2 (StataCorp, TX, USA).

## RESULTS

Over an average follow-up of 7 years, we identified a total of 3714 incident cancers (263 533 person-years) and 1153 cancer deaths (272 425 person-years). Baseline characteristics of the study population by quartiles of total PA are shown in Table 1. Compared with men in the lowest quartile of total PA (<38 MET-h day^−1^), those in the highest quartile were more likely to be never alcohol drinkers and never smokers, and were less likely to have post-secondary education and history of diabetes.

**Figure 1:**
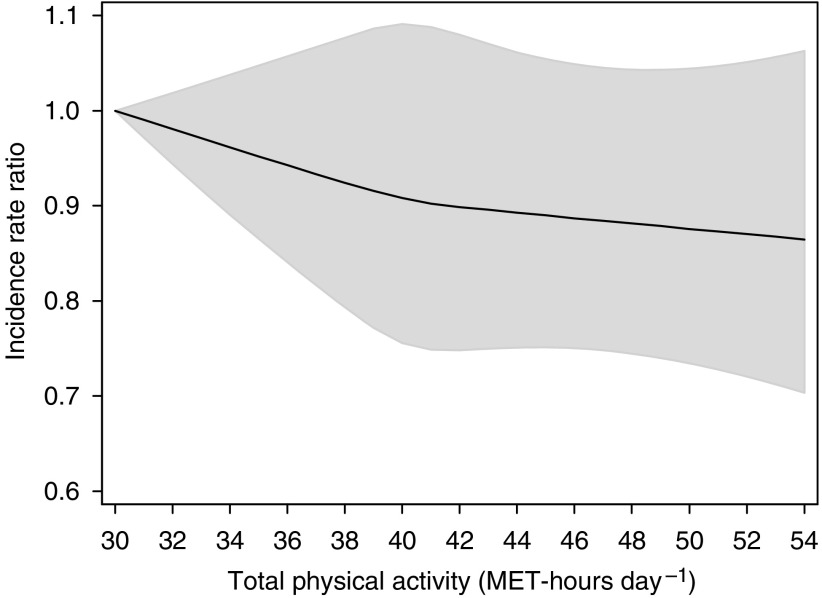
Multivariate incidence rate ratios for cancer in all sites according to total physical activity, expressed by MET-h day^−1^. Data were fitted using a restricted cubic spline Cox regression model adjusted for age (continuous), body mass index (continuous), smoking status and pack-years of smoking (never, former <20 pack-years, former 20–39 pack-years, former ⩾40 pack-years, current <20 pack-years, current 20–39 pack-years, current ⩾40 pack-years), alcohol consumption (current drinker, former drinker, never drunk), educational level (less than high school, high school graduate, and more than high school), history of diabetes (yes, no), and parental history of cancer (yes, no, not known). Solid curve represents point estimates and grey shaded area denotes 95% confidence bands.

Figure 1 shows incidence RR by total PA levels. The age-adjusted incidence RR for every 4 MET-h day^−1^ increment of total PA, which is approximately equivalent to 1 h of moderate effort, was 0.96 (95% CI=0.93–1.00). Further adjustment for BMI, smoking and pack-years, alcohol, education, diabetes, and parental history of cancer slightly attenuated the estimate; each 4 MET-h day^−1^ increment of PA was associated with an incidence RR of 0.98 (95% CI=0.94–1.01). The test for interaction between smoking status and total PA in predicting cancer was not significant (*P*=0.83).

**Figure 2:**
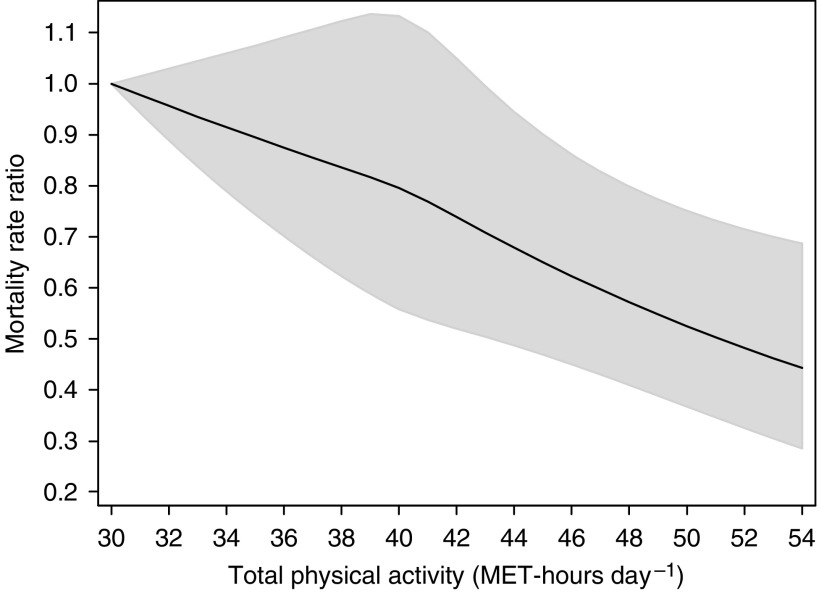
Multivariate mortality rate ratios for death from cancer in all sites according to total physical activity, expressed by MET-h day^−1^. Data were fitted using a restricted cubic spline Cox regression model adjusted for age (continuous), body mass index (continuous), smoking status and pack-years of smoking (never, former <20 pack-years, former 20–39 pack-years, former ⩾40 pack-years, current <20 pack-years, current 20–39 pack-years, current ⩾40 pack-years), alcohol consumption (current drinker, former drinker, never drunk), educational level (less than high school, high school graduate, and more than high school), history of diabetes (yes, no), and parental history of cancer (yes, no, not known). Solid curve represents point estimates and grey shaded area denotes 95% confidence bands.

Total PA was statistically significantly inversely associated with cancer mortality RRs (MRRs) (Figure 2). The spline curve showed an inverse linear dose-response relationship with cancer mortality, and there was no evidence of departure from linearity (*P*=0.41). The multivariate-adjusted MRR in the top quartile (>43 MET-h day^−1^) of total PA was 0.71 (95% CI=0.58–0.88) as compared to the lowest quartile (<38 MET-h day^−1^). The highest level of total PA (54 MET-h day^−1^) was associated with 54% (95% CI=30–70%) lower death rate from cancer compared to those with the lowest level (30 MET-h day^−1^).

Age and multivariate-adjusted cancer MRRs corresponding to defined increments of total PA (4 MET-h day^−1^) based on complete case and multiple imputation analyses are shown in Table 2, age-adjusted MRR being associated with 14% (95% CI=8–20%) lower cancer death rate. Further adjustment for BMI, smoking, alcohol, education, diabetes, and parental history of cancer did not substantially change the MRR; each increment being associated with 12% (95% CI=6–18%) lower cancer death rate.

**Figure 3:**
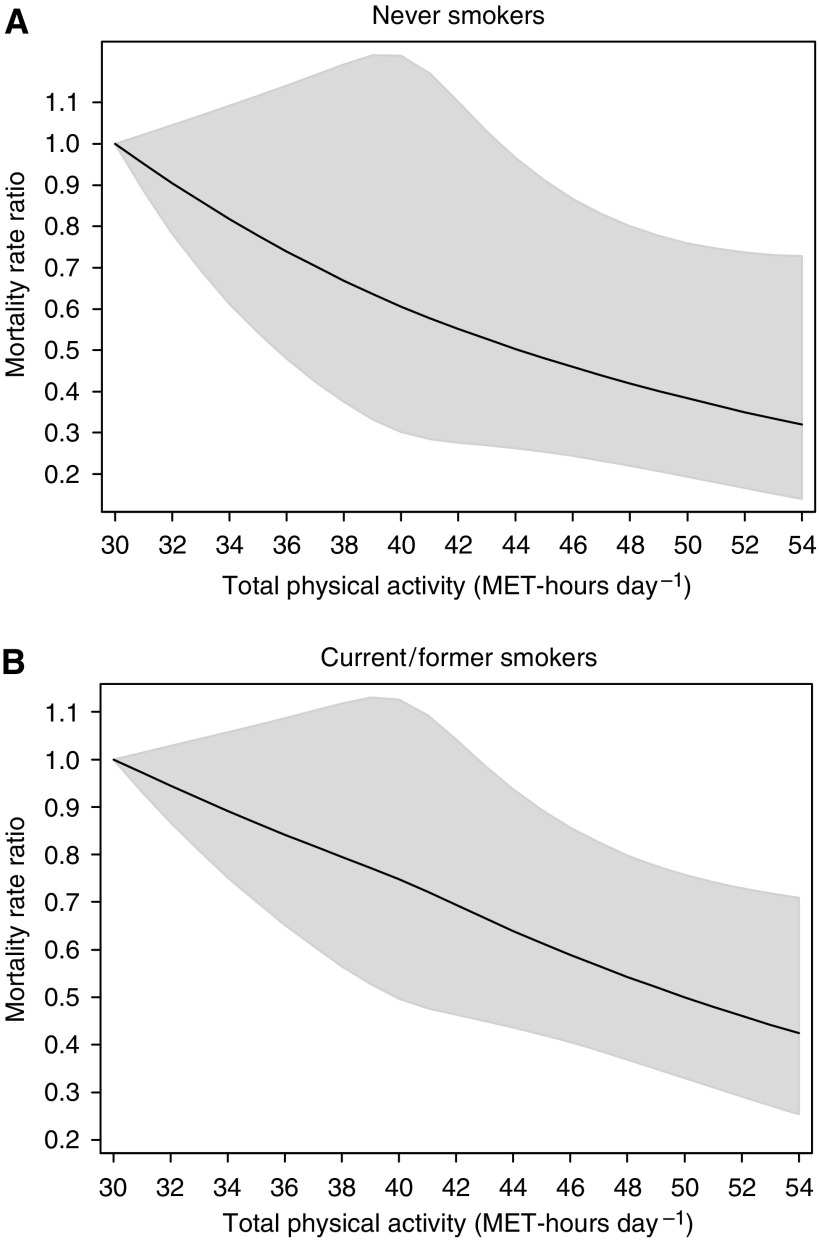
Multivariate mortality rate ratios for death from cancer in all sites according to total physical activity, expressed by MET-h day^−1^, among never (**A**) and current/former smokers (**B**). Data were fitted using a restricted cubic spline Cox regression model adjusted for age (continuous), body mass index (continuous), alcohol consumption (current drinker, former drinker, never drunk), educational level (less than high school, high school graduate, and more than high school), history of diabetes (yes, no), and parental history of cancer (yes, no, not known). Solid curve represents point estimates and grey shaded area denotes 95% confidence bands.

To assess whether the observed inverse relationship with PA reflected reverse causation due to smoking, we stratified the analysis by smoking status (Table 2), but no variation was found (*P* interaction=0.48) as shown graphically in Figure 3 comparing never *vs* current and former smokers.

Both the magnitude and direction of the MRRs based on complete information and multiple imputation analyses were overall very similar (Table 2); the average of the relative differences ((complete case − multiple imputation)/multiple imputation) was 2% (range from 1 to 6%). To examine whether preclinical symptoms influenced PA, thereby biasing our results, we excluded cases in the first 2 years of follow-up. The main results did not change substantially; the MRRs for 4 MET-h day^−1^ increase of total PA were 0.90 (95% CI=0.83–0.97). We also cross-classified participants by age (⩽65 *vs* >65 years) and BMI (<25 *vs* 25 kg/m^2^), but no significant interaction with age (*P*=0.51), or BMI (*P*=0.09) emerged.

**Figure 4:**
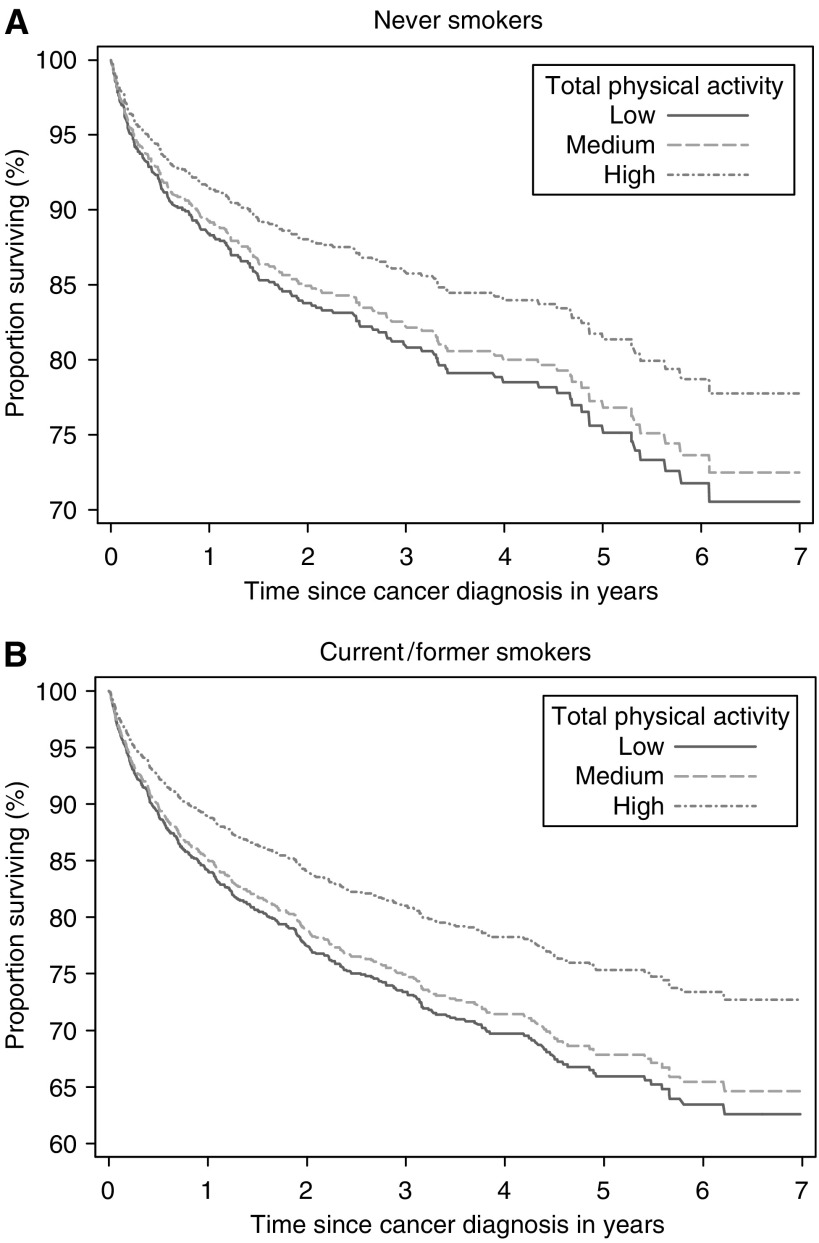
Cumulative survivor function according to levels of total physical activity (low=bottom quartile; medium=interquartile range; high=top quartile) among never (**A**) and current/former smokers (**B**). Survival probabilities after cancer diagnosis, expressed as percentages, were estimated using a Cox regression model adjusted for age (continuous), body mass index (continuous), alcohol consumption (current drinker, former drinker, never drunk), educational level (less than high school, high school graduate, and more than high school), history of diabetes (yes, no), and parental history of cancer (yes, no, not known).

Overall, men in the top quartile of total PA had higher cancer survival (MRR=0.69, 95% CI=0.53–0.89) than those in the lowest quartile throughout follow-up, being 77% for men in the top quartile, >43 MET-h day^−1^, which was significantly higher than 70% for medium (interquartile range, 38–43 MET-h day^−1^) activity levels and 69% for low (bottom quartile, <38 MET-h day^−1^) activity levels. There was no evidence that these relationships varied for smoking status (*P* interaction=0.95) as shown in Figure 4. In addition, no significant effect modification by time since diagnosis was observed (*P* interaction=0.34).

Compared to men who hardly ever walked or biked, walking or bicycling an average of 30 min day^−1^ was associated with a nonsignificant trend for a 5% reduction in cancer incidence. An increased duration of walking or bicycling, ranging from 60 to 90 min day^−1^, was associated with a significant 16% reduction (95% CI=2–28%) in cancer incidence (Table 3). Compared to men who hardly ever walked or biked, walking or bicycling an average of 30 min day^−1^ was associated with a statistically significant 34% (95% CI=18–47%) reduction in cancer mortality in the analytic cohort and a statistically significant 33% (95% CI=14–47%) improvement in cancer survival.

## DISCUSSION

In this large cohort study of men, we observed a statistically significant strong inverse dose-response association between total daily PA and cancer mortality. Total daily PA corresponding to 1 h of moderate effort was associated with 12% decrease in cancer mortality. Overall, men in the top PA quartile had higher survival than to those in the lowest quartile, 5-year cancer survival being 77% for high levels at PA and 69% for low levels. The main component of active living, walking or bicycling 30 min day^−1^, was associated with a significant 34% lower mortality compared to walking or biking hardly ever. Furthermore, cancer survival was improved by 33% among those who walk or biked 30 min day^−1^. Association of PA with cancer incidence was weak, though, walking or bicycling at least 60 min day^−1^ was associated with a significant 16% reduction in cancer incidence.

Linear decrease in cancer mortality with total PA accords with an American prospective study among 252 925 participants (142 828 men) aged 50–71 years, which observed a significant (*P* for trend=0.02) inverse relationship between cancer mortality and the number of hours per week of activity of at least moderate intensity (Leitzmann *et al*, 2007). Similar to our study, no effect modification was observed by smoking, age or BMI. With respect to the recommendation of 30 min day^−1^ of moderate PA on most days, we found a 34% reduction compared to a 17% reduction in the US study (Leitzmann *et al*, 2007), a difference perhaps due to different PA assessments.

The finding that men in the top PA quartile had 29% lower cancer mortality accords with a previous study, which during 17.7 years of follow-up (2039 cancer deaths) observed a significant protective effect (20%) of combined leisure-time and occupational PA on cancer mortality comparing the highest *vs* the lowest PA category (Hu *et al*, 2005), but assessed only qualitatively (low, medium, high). In contrast, we modelled PA as a continuous quantitative score and found an inverse linear dose-response trend without a plateau, implying that there is no threshold above which the beneficial effect of PA ceases.

We found a nonsignificant inverse association between walking and bicycling for 30 min day^−1^ and cancer incidence, though with longer duration, of at least 60 min was significantly associated with 16% lower risk of cancer. This finding supports the PA recommendation of the World Cancer Research Fund/American Institute for Cancer Research which calls for moderate activity (incorporated in occupational, household, or leisure-activities) of 60 min day^−1^ or more (WCRF/AICR, 2007).

Our results may be explained, biologically, through improvement of insulin resistance and increased adiponectin levels, both of which are associated with decreased cancer risk (Kelesidis *et al*, 2006; Bluher *et al*, 2007). Exercise decreases insulin resistance and thus circulating insulin levels (Goodyear and Kahn, 1998).

Major strengths of our study include its large size, its population-based and prospective design, the relatively large number of cancers, and the completeness of case ascertainment, minimising potential recall and selection biases, and, improving the generalisability of our findings.

Although a potential limitation is that PA was assessed through a self-administered questionnaire, with possible classification errors, our validation study was reassuring. Moreover, since information on exposures was collected prospectively, any non-differential misclassification would most likely have attenuated any true relationships and is unlikely to explain the observed associations.

The proportional hazards regression model may lead to substantial biased estimates when missing covariate data are dependent on outcome or both the outcome and the exposure (Demissie *et al*, 2003). In our study the small differences, however, between complete case and multiple imputation analyses suggested that any missing data was random.

An additional concern is that the observed associations might reflect residual confounding by smoking and reverse causation, if heavy smokers dying during the follow-up were unable to be physically active. However, adjusting for many factors as well as exclusion of the first 2-years of follow-up did not alter the risk estimates appreciably, thus suggesting that uncontrolled or residual confounding is unlikely to explain our findings. Moreover, regardless of the end point (incidence, mortality, or survival), we found no evidence of effect modification by smoking status. The beneficial effects on cancer survival may reflect reverse causation, if low PA resulted from an under diagnosed cancer with a relatively short survival. If this were true, an interaction between PA and time since diagnosis might be apparent, but no evidence of this was found. Our findings, which may have major public health implications in the prevention and treatment of malignancies, require confirmation.

## Figures and Tables

**Table 1:** Age-standardised baseline characteristics by quartiles of total physical activity in the cohort of Swedish men aged 45–79 years, followed-up 1998–2004

	**Quartiles of total physical activity, range (median), MET-h per day^a^**				
**Characteristics^b^**	**<38 (36)**	**38–40.0 (39)**	**40–43 (42)**	**>43 (45)**	**Missing**
No. of individuals	7662	7663	7663	7662	10058
No. of cancer diagnosis^c^	769	692	797	469	987
No. of cancer deaths^c^	217	181	197	185	373
Mean age at baseline, year	60	59	60	62	62
Mean body mass index, kg m^−2^	26	26	26	25	26
Postsecondary education, %	42	40	32	23	25
Never alcohol drinker, %	4	5	5	6	6
Never smoker, %	34	38	38	38	33
History of diabetes, %	10	9	8	7	13
Family history of cancer, %	44	44	44	44	47

a MET=metabolic equivalent.

b All factors (except age) were directly standardised to the age distribution of the entire study cohort (*n*=40 708). Values presented are percentages unless indicated otherwise.

c Based on International Classification of Diseases (ICD-10, codes C00-C97).

**Table 2:** Age-adjusted and multivariate-adjusted cancer mortality rate ratios according to increments of total daily physical activity among all men, never smokers, and current or former smokers

	**MRR (95% CI) per 4 MET-h per daya,^b^**		
**Cancer mortality**	**Age-adjusted^c^**	**Multivariate-adjustedc,^d^**	**Multiple imputationc,^e^**
*Total physical activity*			
No. of subjects	*n*=28 880	*n*=28 880	*n*=40 708
All men	0.86 (0.80–0.92)	0.88 (0.82–0.94)	0.89 (0.84–0.95)
By smoking status			
Never	0.84 (0.74–0.95)	0.83 (0.72–0.95)	0.88 (0.79–0.97)
Current and former	0.87 (0.81–0.95)	0.90 (0.83–0.97)	0.89 (0.83–0.97)

a CI=confidence interval; MRR=mortality rate ratios.

b MET=metabolic equivalent, 4 MET correspond to physical activities of moderate effort for 1 h day^−1^.

c MRRs were adjusted for age (continuous), body mass index (continuous), smoking status and pack-years of smoking (never, former <20 pack-years, former 20–39 pack-years, former ⩾40 pack-years, current <20 pack-years, current 20–39 pack-years, current ⩾40 pack-years), alcohol consumption (current drinker, former drinker, never drunk), educational level (less than high school, high school graduate, and more than high school), history of diabetes (yes, no), and parental history of cancer (yes, no, not known).

d Complete case analysis discarded missing values on any covariate.

e Multiple imputation analysis based on five imputed data sets of the analytic cohort and estimates combined using Rubin's method.

**Table 3:** Percentage of reduction in cancer incidence and mortality in the analytic cohort, and percentage of improvement in survival in the subset of men diagnosed with cancer in relation to daily walking or bicycling

	**Walking/bicycling**	
**Cancer**	**Hardly ever**	**Active**
	Percentage reduction (95% CI)^a^	
	*n*=28 880	
		
Incidence for 60–90 min day^−1^	Referent	16% (2–28%)
Mortality for 30 min day^−1^	Referent	34% (18–47%)
		
	Percentage improvement (95% CI)^a^	
	*n*=2551	
		
Survival after cancer diagnosis for 30 min day^−1^	Referent	33% (14–47%)

a CI=confidence interval. The percentage is calculated from the multivariate rate ratio (RR) and its CI as (1-RR) × 100. The RRs were adjusted for age (continuous), body mass index (continuous), smoking status and pack-years of smoking (never, former <20 pack-years, former 20–39 pack-years, former ⩾40 pack-years, current <20 pack-years, current 20–39 pack-years, current ⩾40 pack-years), alcohol consumption (current drinker, former drinker, never drunk), educational level (less than high school, high school graduate, and more than high school), history of diabetes (yes, no), and parental history of cancer (yes, no, not known).
